# Esaxerenone Attenuates Aldosterone-Induced Renal Fibrosis by Suppressing Fibroblast-to-Lymphatic Endothelial-like Cell Transdifferentiation

**DOI:** 10.3390/ijms27104297

**Published:** 2026-05-12

**Authors:** Jie Wang, Sijia Yang, Xiaoheng Wang, Yi Chang, Fan Yang, Panpan Qiang, Xiangting Wang, Tatsuo Shimosawa, Qingyou Xu, Yunzhao Xiong

**Affiliations:** 1 Graduate School, Hebei University of Chinese Medicine, Shijiazhuang 050200, China; 2 Hebei Key Laboratory of Integrative Medicine on Liver-Kidney Patterns, Hebei University of Chinese Medicine, Shijiazhuang 050200, China; 3 College of Integrative Medicine, Hebei University of Chinese Medicine, Shijiazhuang 050200, China; 4 Department of Clinical Laboratory, School of Medicine, International University of Health and Welfare, Narita 286-8686, Japan

**Keywords:** aldosterone, fibroblasts, lymphangiogenesis, mineralocorticoid receptor, VEGFC, VEGFR-3, esaxerenone

## Abstract

Fibroblasts and lymphangiogenesis promote renal fibrosis. However, whether fibroblasts promote renal fibrosis via lymphangiogenesis has not yet been fully elucidated. This study set out to clarify whether aldosterone induces fibroblast transdifferentiation into lymphatic endothelial-like cells, thus promoting lymphangiogenesis and renal fibrosis. In vivo, twenty-four male Wistar rats were randomized into Sham, ALD (6-week aldosterone infusion), and ALD + ESA (aldosterone infusion with esaxerenone co-treatment) groups. In vitro, primary rat renal interstitial fibroblasts (RKFs) were used. Immunohistochemistry and Western blot were used to detect lymphatic endothelial and fibroblast marker expression in kidneys from aldosterone-infused rats and RKFs. Their co-expression was determined by flow cytometry and immunofluorescence co-staining. Mineralocorticoid receptor (MR) activation and related signaling pathways were also analyzed by Western blot, immunohistochemistry, and flow cytometry. Additionally, RKF migration and tube formation were examined to investigate the role of aldosterone-induced fibroblast-to-lymphatic endothelial-like transdifferentiation in renal fibrosis. Our data suggest that aldosterone activates the MR and induces the transdifferentiation of fibroblasts into lymphatic endothelial-like cells via the MR/VEGFC/VEGFR-3 pathway, thereby promoting lymphangiogenesis. In addition, the administration of esaxerenone (a mineralocorticoid receptor blocker, MRB) to rats significantly suppresses this transdifferentiation and alleviates fibrosis.

## 1. Introduction

Inflammatory lymphangiogenesis and related infiltration accelerate the progression process of renal fibrosis [1,2]. By modulating intrarenal immune and inflammatory responses, lymphangiogenesis may further promote renal structural abnormalities and fibrosis [3]. In diverse fibrosis models as well as chronic kidney disease (CKD) patients complicated with multiple pathologies, prominent lymphangiogenesis occurs at renal interstitial lesion sites [4]. It is widely accepted that the majority of newly generated lymphatic vessels derive from resident lymphatic endothelial cells; however, Gang Xu et al. found that M1 macrophages facilitate lymphatic vessel formation in the microenvironment of renal fibrosis [5]. Fibroblasts can secrete inflammatory factors, thereby promoting the progression of the inflammatory process [6]. In addition, numerous studies have confirmed that fibroblasts display remarkable plasticity and multilineage capacity, with the ability to undergo transdifferentiation into various mesenchymal or non-mesenchymal cell lineages under certain conditions. Renal resident fibroblasts are capable of converting into myofibroblasts through epithelial–mesenchymal transition (EMT) and endothelial–mesenchymal transition (EndMT), and directly drive the pathogenesis of renal fibrosis via the secretion of extracellular matrix [7]. In addition, fibroblasts can differentiate into adipocytes, chondrocytes, and osteoblast-like cells [8]. We previously reported that fibroblasts can transdifferentiate into macrophage-like cells during renal inflammation [9], and that inflammation-mediated macrophage transdifferentiation into lymphatic endothelial cells drives lymphangiogenesis [10], ultimately promoting the development of renal fibrosis. Whether fibroblasts can transform into lymphatic endothelial-like cells remains unknown.

In this study, we investigated chronic renal fibrosis induced by aldosterone infusion in rats to explore whether renal mineralocorticoid receptor (MR) activation is involved in the transdifferentiation of renal fibroblasts into lymphatic endothelial-like cells, thereby promoting interstitial fibrosis and ultimately contributing to the development of CKD.

## 2. Results

### 2.1. Esaxerenone Significantly Reduces Renal Fibrosis in Aldosterone-Infused Rats

**Figure 1:**
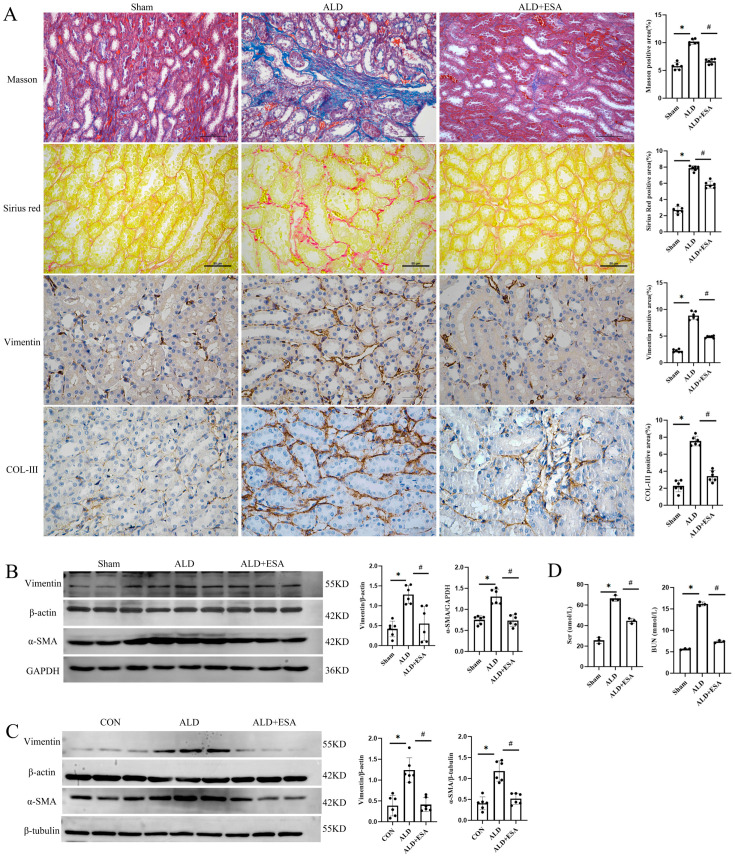
Esaxerenone significantly reduces renal fibrosis in aldosterone-infused rats. (**A**) Masson staining, Sirius red staining, and Vimentin/COL-III immunohistochemistry were performed to detect pathological changes and fibrosis in rat kidneys (*n* = 6); scale bars, 100 (Masson) and 50 (others). All in µm. (**B**) Western blot was used to determine the protein expression levels of α-SMA and Vimentin in rat kidneys (*n* = 6). (**C**) Western blot was employed to measure the protein expression levels of α-SMA and Vimentin in RKFs after aldosterone stimulation (*n* = 6). (**D**) Serum creatinine and blood urea nitrogen were detected for renal function evaluation (*n* = 3). All data are presented as mean ± SD, and one-way ANOVA was used to examine intergroup differences (* *p* < 0.05 vs. Sham group; ^#^ *p* < 0.05 vs. ALD group).

This study centered on chronic renal injury in aldosterone-infused rats; accordingly, kidneys were harvested 6 weeks after subcutaneous aldosterone infusion. Masson staining showed extensive collagen accumulation and fibrotic lesions in the kidneys of the ALD group in comparison with the Sham group (Figure 1A). Concordantly, Sirius red staining indicated elevated collagen deposition in the ALD group (Figure 1A). Immunohistochemical (IHC) staining for Vimentin and collagen III (COL-III) in renal sections further supported these findings (Figure 1A).

To further evaluate renal fibrosis in aldosterone-infused rats, Western blot analysis of Vimentin and α-SMA expression in kidneys was performed, showing significantly higher protein levels in the ALD group than in the Sham group, and this response was reversed by esaxerenone (Figure 1B). Subsequent Western blot analysis of RKFs confirmed that the expression levels of Vimentin and α-SMA were significantly higher in the ALD group than in the CON group (Figure 1C), consistent with our previous results. In agreement with the pathological changes, serum creatinine (Scr) and blood urea nitrogen (BUN) measurements verified renal dysfunction in aldosterone-infused rats (Figure 1D).

### 2.2. Esaxerenone Attenuated the Transdifferentiation of Renal Fibroblasts into Lymphatic Endothelial-like Cells in Aldosterone-Infused Rats

**Figure 2:**
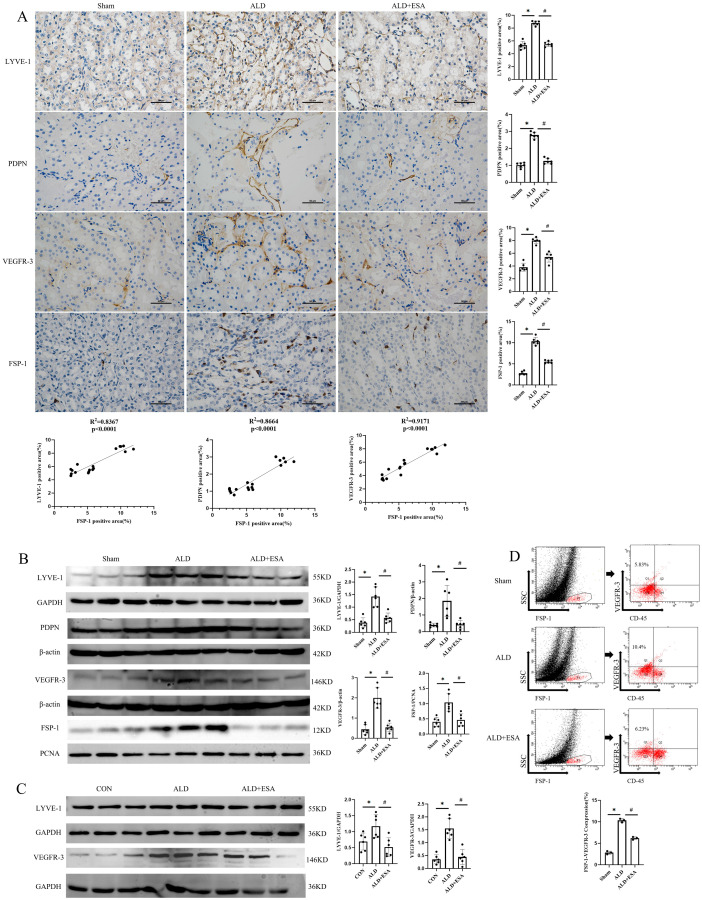
Esaxerenone attenuated the transdifferentiation of renal fibroblasts into lymphatic endothelial-like cells in aldosterone-infused rats. (**A**) Immunohistochemistry staining using antibodies against LYVE-1, PDPN, VEGFR-3 and FSP-1 to examine transdifferentiation of renal fibroblasts into lymphatic endothelial-like cells in the rat kidneys (*n* = 6); scale bars, 50 μm. The correlation of FSP-1 expression with the positive expressions of LYVE-1, PDPN, and VEGFR-3 was evaluated. (**B**) Western blot was employed to examine the protein levels of LYVE-1, PDPN, VEGFR-3, and FSP-1 in the rat kidneys, followed by quantitative analysis (*n* = 6). (**C**) Western blot was performed to determine the protein expressions of LYVE-1 and VEGFR-3 in RKFs after aldosterone stimulation, with subsequent quantitative analysis (*n* = 6). (**D**) The expressions of FSP-1 and VEGFR-3 in the kidney were analyzed by flow cytometry. First, all fibroblasts in the rat kidney were identified, and Q1 represents the proportion of fibroblasts that transdifferentiated into lymphatic endothelial-like cells among the non-immune cells (*n* = 3). All data are presented as mean ± SD, and one-way ANOVA was used to examine intergroup differences (* *p* < 0.05 vs. Sham group; ^#^ *p* < 0.05 vs. ALD group).

Previous research has revealed a strong link between organ fibrosis and abnormalities in the intrinsic lymphatic system [4]. To elucidate the interplay between fibroblasts and lymphangiogenesis in renal fibrosis, we conducted IHC staining on kidneys from aldosterone-infused rats using antibodies against LYVE-1, PDPN, and VEGFR-3 (specific lymphangiogenic markers) as well as FSP-1 (a fibroblast-specific marker). We observed a substantial accumulation of fibroblasts and enhanced lymphangiogenesis in the kidneys of these rats, and these effects were also inhibited by esaxerenone treatment (Figure 2A). Subsequently, correlation analysis was performed on the IHC staining results. The expression of FSP-1 was positively linearly correlated with the density of LYVE-1 (R^2^ = 0.8367, *p*  <  0.0001), PDPN (R^2^ = 0.8664, *p* < 0.0001), and VEGFR-3 (R^2^ = 0.9171, *p*  <  0.0001) (Figure 2A). Western blot analysis of kidneys in aldosterone-infused rats (Figure 2B) and RKF (Figure 2C) also showed that lymphangiogenic markers were upregulated along with the increased activation of fibroblasts. To further investigate the fibroblasts–lymphangiogenesis relationship, aldosterone-infused rats were used to harvest kidney cells for flow cytometric analysis. After regular gating, FSP-1 was used to identify fibroblasts, and the percentage of VEGFR-3^+^ cells was examined in CD45^−^ fibroblasts. The proportion of FSP-1^+^VEGFR-3^+^ cells was 5.83% in the Sham group, which significantly increased to 10.4% in the ALD group, and decreased to 6.23% after esaxerenone treatment (Figure 2D).

**Figure 3:**
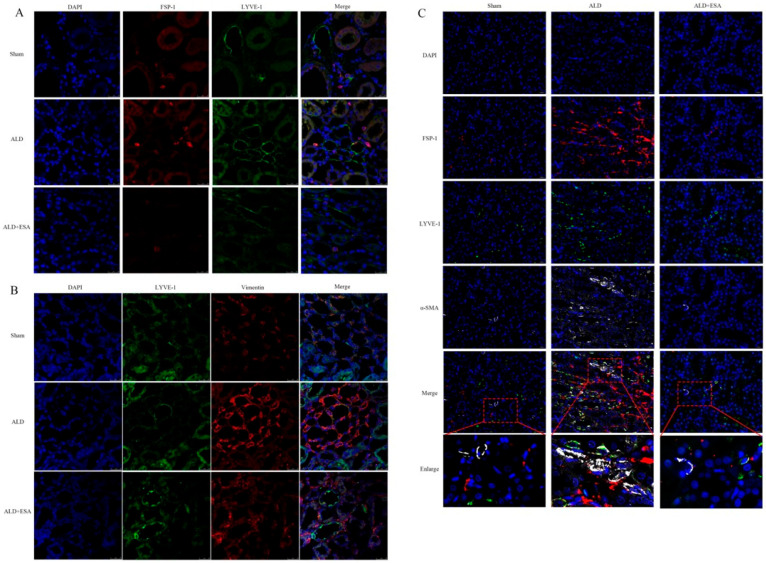
Rat renal lymphatic endothelial cells co-express fibroblast and fibrosis markers. (**A**) Immunofluorescence staining of rat kidneys was conducted using antibodies against FSP-1 (red) and LYVE-1 (green); cell nuclei were stained with DAPI (blue). (**B**) Immunofluorescence staining of rat kidneys was carried out with antibodies against Vimentin (red) and LYVE-1 (green); cell nuclei were stained with DAPI (blue). (**C**) Immunofluorescence staining of rat kidneys was performed using antibodies against FSP-1 (red), LYVE-1 (green), and α-SMA (white); cell nuclei were stained with DAPI (blue).

Meanwhile, immunofluorescent co-staining for FSP-1 and LYVE-1 was performed. As shown in Figure 3A, most LYVE-1^+^ cells in the ALD group co-expressed FSP-1, and this co-localization was markedly reduced by esaxerenone treatment. To determine whether lymphangiogenesis contributes to renal fibrosis in aldosterone-infused rats, we performed immunofluorescent co-staining for Vimentin and LYVE-1. As shown in Figure 3B, colocalization of Vimentin and LYVE-1 was stronger in the ALD group than in the Sham and ALD + ESA groups. Next, aldosterone-infused rat kidneys were stained with antibodies against FSP-1, LYVE-1, and α-SMA. As expected, FSP-1^+^LYVE-1^+^ cells were surrounded by α-SMA, indicating that fibroblasts can transdifferentiate into lymphatic endothelial-like cells and produce α-SMA (Figure 3C).

### 2.3. Esaxerenone Inhibits the Transdifferentiation of Fibroblasts into Lymphatic Endothelial-like Cells by Modulating the MR/VEGFC/VEGFR-3 Signaling Pathway

**Figure 4:**
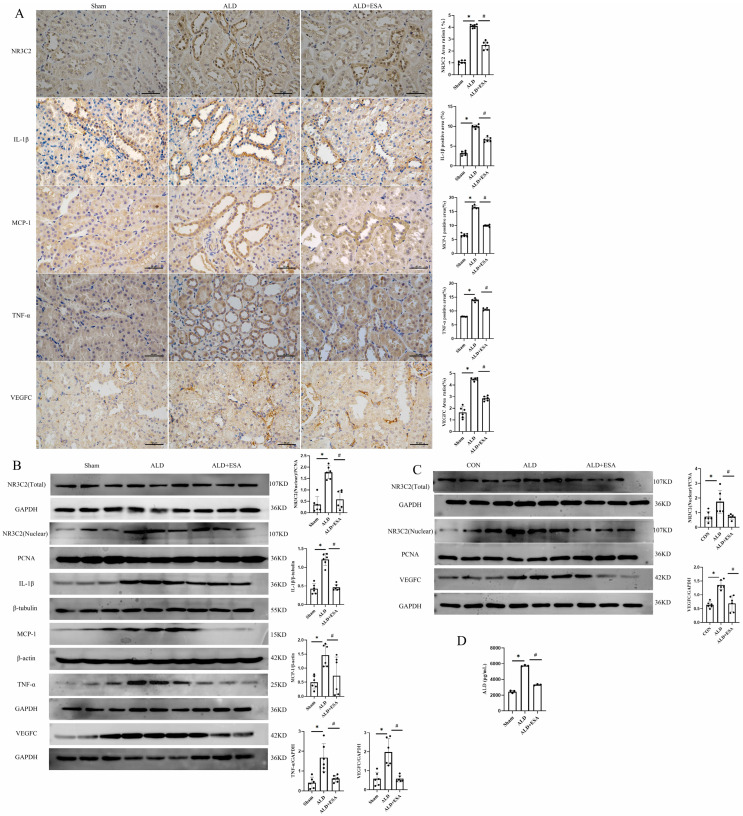
MR activation and inflammatory factors changes in aldosterone-infused rats. (**A**) Immunohistochemistry was used to detect the expression of NR3C2, IL-1β, MCP-1, TNF-α, and VEGFC in rat kidneys (*n* = 6); scale bars, 50 μm. (**B**) Western blot was performed to determine the protein expression levels of NR3C2, IL-1β, MCP-1, TNF-α, and VEGFC in rat kidneys (*n* = 6); (**C**) Western blot was used to detect the protein expression of NR3C2 and VEGFC in RKFs after aldosterone stimulation (*n* = 6); (**D**) Serum aldosterone detection (*n* = 3). All data are presented as mean ± SD, and one-way ANOVA was used to examine intergroup differences (* *p* < 0.05 vs. Sham group; ^#^ *p* < 0.05 vs. ALD group).

Plasma aldosterone was elevated in the aldosterone-infused rats, which may increase MR activity. In this study, we confirmed renal MR activation in these rats by detecting MR downstream molecules using IHC and Western blot. Specifically, IHC demonstrated significant upregulation of NR3C2, IL-1β, MCP-1, TNF-α, and VEGFC (a lymphangiogenesis-related marker) in the ALD group, with esaxerenone effectively reversing these upregulatory effects (Figure 4A). Consistent with the IHC results, the expression levels of NR3C2, PCNA, IL-1β, MCP-1, TNF-α and VEGFC were notably elevated in the ALD group compared to the Sham group (Figure 4B), which was indicative of increased renal MR activity; as expected, this pro-inflammatory response was suppressed by the MR blocker esaxerenone. To further investigate the role of MR activation in the transdifferentiation of fibroblasts into lymphatic endothelial-like cells, we examined the effects of aldosterone and MR blockade on NR3C2 and VEGFC expression in RKF. Aldosterone significantly increased the protein expression of these two molecules in RKF, an effect that was blocked by esaxerenone (Figure 4C). Enzyme-linked immunosorbent assay (ELISA) demonstrated that serum aldosterone levels were significantly higher in the ALD group than in the Sham group, and esaxerenone attenuated this increase (Figure 4D).

**Figure 5:**
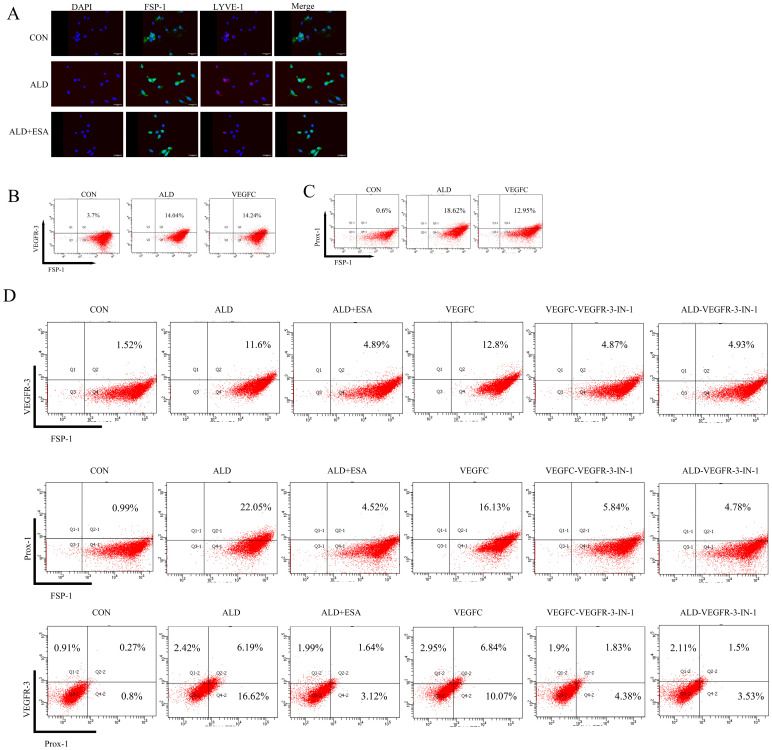
Esaxerenone inhibits fibroblast transdifferentiation into lymphatic endothelial-like cells via the MR/VEGFC/VEGFR-3 signaling pathway. (**A**) Immunofluorescence staining of RKFs was performed using antibodies against FSP-1 (green) and LYVE-1 (red); cell nuclei were stained with DAPI (blue); scale bars, 100 μm. (**B**) Flow cytometry was used to analyze the co-expression of FSP-1 and VEGFR-3 in RKFs after stimulation with aldosterone or VEGFC (*n* = 3). (**C**) Flow cytometry was used to analyze the co-expression of FSP-1 and Prox-1 in RKFs after stimulation with aldosterone or VEGFC (*n* = 3). (**D**) Flow cytometry was employed to detect the co-expression of FSP-1 and VEGFR-3, FSP-1 and Prox-1, as well as Prox-1 and VEGFR-3 in RKFs (*n* = 3).

In vitro, we performed immunofluorescent co-staining for FSP-1 and LYVE-1 in RKFs. As shown in Figure 5A, the number of FSP-1^+^LYVE-1^+^ double-positive cells was significantly increased in the ALD group, and esaxerenone markedly suppressed this co-expression. Consistent with the immunofluorescent results, aldosterone or VEGFC stimulation upregulated VEGFR-3 and Prox-1 protein expression in RKFs (Figure 5B,C), and this effect was attenuated by esaxerenone or VEGFR-3-IN-1 (a specific VEGFR-3 inhibitor). Additionally, the number of FSP-1^+^VEGFR-3^+^, FSP-1^+^Prox-1^+^, and Prox-1^+^VEGFR-3^+^ double-positive RKFs was lower in the ALD + VEGFR-3-IN-1 group than in the ALD group, indicating that VEGFR-3 inhibition suppresses aldosterone-induced lymphangiogenesis (Figure 5D).

**Figure 6:**
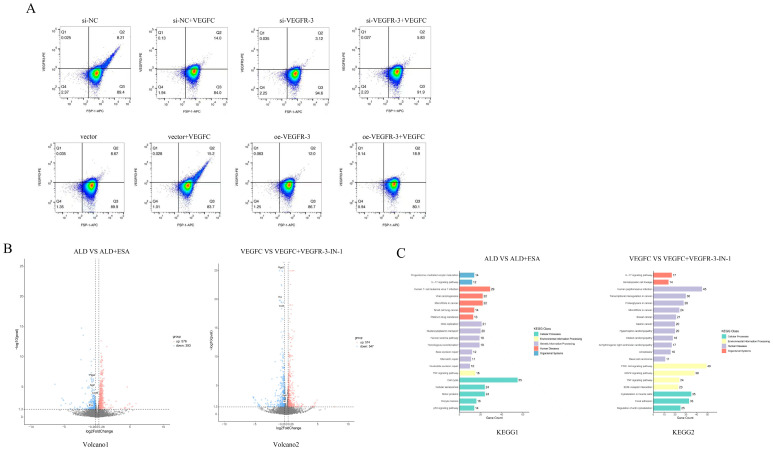
Effects of VEGFR-3 knockdown/overexpression on phenotypic transformation of RKFs and transcriptomic analysis. (**A**) Flow cytometry was used to analyze the co-expression of FSP-1 and VEGFR-3 in RKFs with siRNA-mediated VEGFR-3 knockdown. Q2 represents the proportion of fibroblasts that transdifferentiated into lymphatic endothelial-like cells (*n* = 3). Flow cytometry was used to analyze the co-expression of FSP-1 and VEGFR-3 in RKFs with VEGFR-3 overexpression. Q2 represents the proportion of fibroblasts that transdifferentiated into lymphatic endothelial-like cells (*n* = 3). (**B**,**C**) Transcriptomic detection of RKFs (*n* = 3).

To verify the effect of VEGFC and VEGFR-3 on fibroblast transdifferentiation into lymphatic endothelial-like cells, we used siRNA to silence VEGFR-3. VEGFR-3 protein was significantly knocked down in the si-VEGFR-3 group compared with the si-NC group. After VEGFC stimulation, the number of FSP-1^+^VEGFR-3^+^ double-positive RKFs was markedly increased, but it remained significantly lower in the si-VEGFR-3 + VEGFC group than in the si-NC + VEGFC group (Figure 6A). We further used flow cytometry to examine the effect of VEGFR-3 overexpression on fibroblast transdifferentiation into lymphatic endothelial like cells. After VEGFR-3 overexpression, oe-VEGFR-3 group RKFs had a significantly higher percentage of FSP-1^+^VEGFR-3^+^ double-positive cells than the vector group. With VEGFC stimulation, oe-VEGFR-3 + VEGFC group had significantly more of these double-positive RKFs than the vector + VEGFC group (Figure 6A).

To further clarify the role of the MR/VEGFC/VEGFR-3 signaling pathway in fibroblast transdifferentiation into lymphatic endothelial-like cells and renal fibrosis, we performed transcriptomic analysis on RKFs. As shown in Figure 6B, compared with the ALD group, intervention with esaxerenone (MRB) significantly downregulated the expression levels of inflammation-related genes such as *Ptgs2*, *Sgk-1*, *Cxcl6*, and *Il1α* in RKFs. In addition, relative to the VEGFC group, treatment with VEGFR-3-IN-1 (VEGFR-3 inhibitor) markedly reduced the expression of inflammatory genes including *Ptgs2*, *Il1α*, *Cxcl6*, *Sgk-1*, and *Il6*. Furthermore, KEGG enrichment analysis revealed significant enrichment of inflammation-associated signaling pathways, such as the IL-17, PI3K-Akt, MAPK, and TNF-α pathways, as well as the fibrosis-related ECM–receptor interaction pathway (Figure 6C).

### 2.4. Effects of Esaxerenone on RKF Migration and Tube Formation In Vitro

While fibroblasts are capable of expressing lymphatic endothelial markers, it remains unclear whether these cells are able to genuinely transdifferentiate into lymphatic vessels. Accordingly, cell migration and tube formation assays were employed to explore this process.

**Figure 7:**
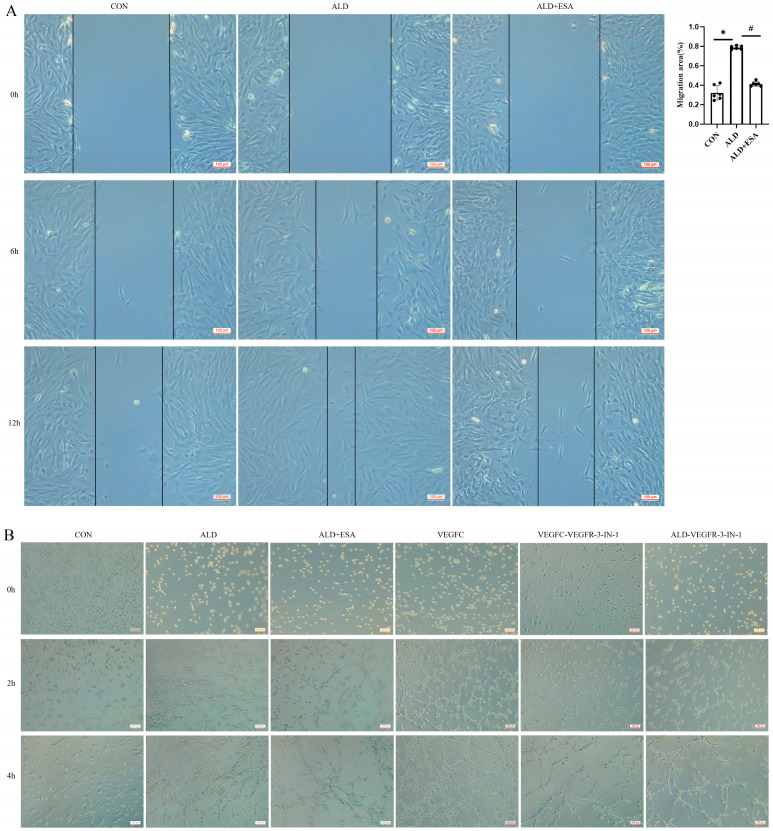
Effects of esaxerenone on RKF migration and tube formation in vitro. (**A**) RKF migration assay (*n* = 3); scale bars, 100 μm. (**B**) RKFs possess a certain migratory capacity in vitro and are able to form three-dimensional tubular structures. The tubular formation was observed and photographed at 0 h, 2 h and 4 h, and qualitative analysis of the network tube length was performed (*n* = 3); scale bars, 100 μm. All data are presented as mean ± SD, and one-way ANOVA was used to examine intergroup differences (* *p* < 0.05 vs. Sham group; ^#^ *p* < 0.05 vs. ALD group).

After scratching the cells in culture dishes, RKFs were stimulated with medium containing ALD and ALD + ESA for 12 h, and images were captured and recorded under a microscope at 0, 6, and 12 h. Findings from the RKF scratch assay demonstrated that the scratch wound healing rate was significantly higher in the ALD group than in the CON group, while this rate was significantly reduced in the ALD + ESA group relative to the ALD group (Figure 7A).

Subsequently, tube formation assays were performed. After stimulation with medium supplemented with aldosterone, VEGFC, esaxerenone, and a specific inhibitor of VEGFR-3, RKFs were initially photographed to record cell status at 0 h, then cultured in an incubator and observed and photographed under a microscope at different time points. The results showed that tube-like structures began to form in the ALD and VEGFC groups at 2 h, whereas no obvious tubular structures were observed in the ALD + ESA, VEGFC + VEGFR-3-IN-1, and ALD + VEGFR-3-IN-1 groups. With prolonged culture time, the number of tube-like structures was significantly increased in the ALD and VEGFC groups. At 4 h, cell clusters in the ALD + ESA, VEGFC + VEGFR-3-IN-1, and ALD + VEGFR-3-IN-1 groups aggregated to form tube-like structures, but their numbers were markedly lower than those in the ALD and VEGFC groups. In contrast, almost no tubular structures formed in the CON group throughout the experiment (Figure 7B). Notably, the ALD + VEGFR-3-IN-1 group formed significantly fewer tubular structures than the ALD group.

## 3. Discussion

Lymphangiogenesis plays a crucial role in maintaining tissue fluid balance, modulating immune function, and promoting lipid uptake. Dysregulated lymphangiogenesis has been associated with various pathological conditions, including malignancies, inflammatory disorders, and autoimmune diseases [11]. While lymphangiogenesis exerts protective effects in removing accumulated fluid and immune cells, renal lymphatics may also trigger an inflammatory feedback loop, thereby aggravating inflammation and fibrosis [12]. Fibroblasts are heterogeneous mesenchymal cells that maintain tissue homeostasis and mediate disease pathogenesis [13]. Their phenotypic transdifferentiation is a key response to injury. Here, this study shows that fibroblasts can transdifferentiate into lymphatic endothelial-like cells, participate in renal lymphangiogenesis, and promote renal fibrosis. MR activation is critical for this transdifferentiation and post-renal injury fibrosis.

Our first finding is that aldosterone can stimulate fibroblasts to transdifferentiate into lymphatic endothelial-like cells, thereby contributing to the progression of renal fibrosis. Fibroblasts exert a pivotal function in this multi-stage pathological process [14]. They are not only involved in the production and accumulation of extracellular matrix, but also release numerous growth factors and cytokines, reshape the renal microenvironment, and impair renal structure and function [15,16]. Previous research has shown that the sustained activation of renal fibroblasts is closely linked to the progression of renal fibrosis, a hallmark of CKD. Additionally, fibroblasts possess plasticity that allows them to transdifferentiate into other cell lineages [17]. Lymphangiogenesis, a critical process in the pathogenesis of renal fibrosis [12], is tightly linked to fibroblast activation. As a mineralocorticoid, aldosterone exhibits potent pro-inflammatory activity in this process [9,10]. By activating fibroblasts and promoting their proliferation and migration, aldosterone contributes to their transdifferentiation into lymphatic endothelial-like cells. Pathological lymphangiogenesis exacerbates renal inflammation, drives fibrosis progression, forms a vicious cycle, disrupts renal physiological homeostasis, and accelerates the progression of CKD [18]. As a mineralocorticoid receptor blocker (MRB), esaxerenone can inhibit the effect of aldosterone [19]. Our findings in Figure 1 and Figure 2 revealed that aldosterone induces fibroblasts to transdifferentiate into lymphatic endothelial-like cells, thus facilitating renal fibrosis, while esaxerenone significantly ameliorates this effect.

Our second finding is that esaxerenone suppresses the transdifferentiation of fibroblasts into lymphatic endothelial-like cells by modulating the MR/VEGFC/VEGFR-3 signaling pathway, thereby attenuating renal fibrosis.

Accumulating evidence suggests that abnormally elevated aldosterone levels are closely associated with the development and progression of various inflammatory diseases [20,21]. Aldosterone activates intracellular signaling pathways through specific receptors and regulates the expression of inflammatory factors, such as MCP-1 and TNF-α. These proteins play a key role in the inflammatory response [9]. MCP-1 recruits immune cells to inflammatory sites, while TNF-α and IL-1β promote inflammatory responses [22]. In the present study, we further confirmed that MR activation contributes to renal fibrosis in aldosterone-infused rats by promoting the transdifferentiation of fibroblasts into lymphatic endothelial-like cells. LYVE-1 is a specific receptor for lymphatic endothelial cells, participating in lymphatic transport and regulating immune and inflammatory responses [23].

VEGFR-3, a receptor tyrosine kinase in vascular and lymphatic endothelial cells, binds specifically to VEGFC and VEGF-D to promote angiogenesis and lymphangiogenesis [24]. Podoplanin (PDPN) is a lymphatic endothelial-specific transmembrane glycoprotein that regulates lymphatic vessel structure and function [25]. VEGFC binds to VEGFR-3 to activate intracellular signaling, thereby promoting angiogenesis and lymphangiogenesis. The VEGFC/VEGFR-3 pathway is closely related to tumor growth, metastasis, and inflammation, and can be induced by tissue injury, interstitial fluid overload, and inflammation [12]. Previous studies have shown that downregulating VEGFC/VEGFR-3 inhibits lymphangiogenesis, reduces LYVE-1 and PDPN expression, and alleviates tubulointerstitial fibrosis and inflammation [26]. In addition to the canonical VEGFC/VEGFR-3 pathway, the Notch signaling pathway [27] and Hippo pathway [28] are also involved in regulating lymphatic endothelial cell differentiation and lymphangiogenesis.

Our data showed that esaxerenone inhibits MR activation and inflammatory mediator secretion, thus blocking fibroblast transdifferentiation into lymphatic endothelial-like cells through the MR/VEGFC/VEGFR-3 pathway. Taken together, our results suggest that activated MR promotes fibroblast transdifferentiation into lymphatic endothelial-like cells, causing excessive collagen deposition and renal fibrosis. MR blockade attenuates this process and protects against renal fibrosis and injury.

To summarize, this study indicates that aldosterone regulates the MR/VEGFC/VEGFR-3 pathway to induce the transdifferentiation of renal fibroblasts into lymphatic endothelial-like cells, which secrete inflammatory factors and mediate renal fibrosis. The MRB esaxerenone can inhibit the aldosterone-induced transdifferentiation of fibroblasts into lymphatic endothelial-like cells, alleviate renal inflammatory injury, and exert a protective effect on the kidneys.

One limitation of this study is that based on triple staining for FSP-1, LYVE-1, and α-SMA, we cannot determine whether α-SMA is produced by fibroblasts or by their transdifferentiated lymphatic endothelial cells. Another limitation is that we mainly focused on non-immune CD45^−^ cells to assess fibroblast activation and transdifferentiation into lymphatic endothelial-like cells during renal fibrosis. However, it remains unclear whether activated immune CD45^+^ cells undergo lymphatic endothelial-like transdifferentiation. Additionally, due to the limitations of available experimental conditions, lineage tracing experiments have not been conducted in this study. Therefore, the current evidence is insufficient to fully confirm that fibroblasts undergo lineage conversion into lymphatic endothelial-like cells. Meanwhile, the direct mechanistic association between the MR/VEGFC/VEGFR-3 signaling pathway and the progression of renal fibrosis remains insufficiently clarified. Furthermore, the direct regulatory effect of MR on VEGFC, as well as whether MR blockade could fully abolish VEGFR-3 activation, were not explored in depth in the present study.

In future research, we will supplement lineage tracing experiments (permanent labeling of fibroblasts) to further verify the lineage conversion of fibroblasts into lymphatic endothelial-like cells. Meanwhile, we will conduct in-depth mechanistic exploration to further clarify the intrinsic relationships among the MR/VEGFC/VEGFR-3 signaling pathway, fibroblast transdifferentiation into lymphatic endothelial-like cells, and renal fibrosis. Moreover, we will elucidate the direct transcriptional regulation and upstream and downstream regulatory networks between MR and VEGFC via promoter assays and targeted intervention experiments.

## 4. Materials and Methods

### 4.1. Animal Model

Twenty-four male *Rattus norvegicus* (Wistar strain, 6 weeks old, body weight 200 ± 20 g) were purchased from Beijing Vital River Laboratory Animal Technology Co., Ltd. (Beijing, China, Animal Production License No.: SCK(Jing)2021-0006, Animal Quality Certificate No.: 110011241105752613). The rats were raised in the Animal Experiment Center of Hebei University of Chinese Medicine, and the specific feeding conditions were as follows: all rats were housed in conventional open breeding cages. The experimental feed was supplied by Beijing Keao Xieli Feed Co., Ltd. (Beijing, China), and animals were given standard animal drinking water. The feeding environment was maintained at a temperature of (24 ± 2) °C, humidity of 40–70%, and a light period from 08:00 to 20:00. All rats could eat and drink freely during the whole experiment. All procedures were approved by the Animal Ethics Committee of Hebei University of Chinese Medicine (Approval No. DWLL202503088). After a minimum of 7 days of acclimation, all rats were randomly divided into 3 groups, with 8 rats in each group. The groups were designated as the Sham group, ALD group (the rats were received continuous aldosterone infusion at 0.75 µg/h via a mini osmotic pump for 6 weeks; CAS NO: 52-39 1, Cayman Chemical, Ann Arbor, MI, USA), and ALD + ESA group (aldosterone infusion + esaxerenone administration via diet at a dosage of 1 mg/kg/day, kindly provided by Daiichi Sankyo Co., Ltd., Tokyo, Japan). After 6 weeks, the rats were initially anesthetized with a low concentration of isoflurane, followed by a rapid transition to a high concentration (5%) to induce immediate loss of consciousness. All animals were sacrificed by excessive anesthesia (specifically, CO_2_ was perfused into the euthanasia chamber at a rate of 10% to 30% of the chamber’s volume per minute. After confirming that the animals were immobile, not breathing, and had dilated pupils, the CO_2_ supply was turned off, followed by a two-minute observation period to confirm their death. Tissues were then collected under a sterile environment).

Additionally, the kidneys were collected for histological and protein analysis, and serum samples from all rats were used to test renal function using BUN (Cat. No.: C013-1-1, Nanjing Jiancheng Bioengineering Institute, Nanjing, China) and Scr (Cat. No.: JL-T0928; ShanghaiJianglai Biotechnology, Shanghai, China) screening kits.

### 4.2. Histological Analysis, Immunohistochemical Analysis, and Immunofluorescence Analysis Pathological Evaluation of Renal Tissue

Kidneys were fixed in 4% paraformaldehyde (PFA) (SEVEN, SI101-01, Beijing, China) overnight, followed by alcohol dehydration, paraffin embedding, and sectioning into 6 μm-thick slices for Masson and Sirius red staining.

ImageJ 2.1.0 software (National Institutes of Health, Bethesda, MD, USA) was used to analyze the positive area ratios in the Masson and Sirius Red stained images.

For immunohistochemical staining, the following antibodies were used: anti-NR3C2 (1:100, ProTeinTech, Chicago, IL, USA; Cat#: 21854-1-AP), anti-IL-1β(1:100, Affinity, Changzhou, China; Cat#: AF4006), anti-MCP-1 (1:50, ProTeinTech, Chicago, IL, USA; Cat#: 26161-1-AP), anti-TNF-α (1:100, Servicebio, Wuhan, China; Cat#: GB11188), anti-VEGFC (1:200, Immunoway, San Jose, CA, USA; Cat#: YT5297), anti-LYVE-1 (1:100, NOVUS, Centennial, CO, USA; Cat#: 2203R01), anti-PDPN (1:400, Boster, Wuhan, China; Cat#: AO1124-2), anti-VEGFR-3 (1:100, Abcam, Shanghai, China; Cat#: AB27878-1001), anti-FSP-1 (1:300, ProTeinTech, Chicago, IL, USA; Cat#: 66489-1-Ig), anti-Collagen-III (1:500, Servicebio, Wuhan, China; Cat#: GB111629), and anti-Vimentin (1:200, Servicebio, Wuhan, China; Cat#: GB11192).

Light images were visualized and captured using a Leica BX53 optical microscope (Leica, Wetzlar, Germany) with a Nikon Digital Sight 10 (Nikon, Tokyo, Japan) and the average positive area ratio (% area) was calculated using ImageJ 2.1.0 software.

For immunofluorescence staining, the kidneys were fixed with 4% PFA, dehydrated in 30% sucrose solution, and embedded in OCT compound for freezing. Next, 7 μm kidney sections were obtained using a freezing microtome and prepared for staining with the following unconjugated antibodies: anti-FSP-1 (1:300, ProTeinTech; Cat#: 66489-1-Ig), anti-LYVE-1 (1:100, NOVUS; Cat#: 2203R01), anti-VEGFR-3 (1:100, Abcam; Cat#: AB27878-1001), anti-Vimentin (1:200, Servicebio; Cat#: GB11192), and anti-α-SMA (1:100, Zenbio, Chengdou, China; Cat#: Z50104) followed by fluorescent secondary staining. After staining, the sections were incubated with DAPI to stain the nuclei and then sealed, followed by photography using a Leica SP8 confocal microscope (CTS SP8; Leica, Wetzlar, Germany).

### 4.3. Protein Extraction and Western Blot Assay

The kidneys were homogenized in RIPA buffer (Bebo Biological, BB-3201, Shanghai, China) to extract total proteins. SDS-PAGE and PVDF membranes were used for Western blot analysis. Following blocking of nonspecific bindings with 5% nonfat milk, the membranes were incubated with primary antibodies against NR3C2 (1:100, ProTeinTech; Cat#: 21854-1-AP), IL-1β (1:100, Affinity; Cat#: AF4006), MCP-1 (1:50, ProTeinTech; Cat#: 26161-1-AP), TNF-α (1:100, Servicebio; Cat#: GB11188), VEGFC (1:200, Immunoway, Suzhou, China; Cat#: YT5297), LYVE-1 (1:100, NOVUS; Cat#: 2203R01), PDPN (1:400, Boster; Cat#: AO1124-2), VEGFR-3 (1:100, Abcam; Cat#: AB27878-1001), FSP-1 (1:300, ProTeinTech; Cat#: 66489-1-Ig), Vimentin (1:200, Servicebio; Cat#: GB11192), and α-SMA (1:100, Zenbio; Cat#: Z50104) were added, and the membrane was incubated overnight at 4 °C. The next day, the blots were subjected to a 1-h incubation with fluorescein-conjugated secondary antibodies at room temperature, followed by scanning with an Odyssey Infrared Imaging System (LI-COR, Lincoln, NE, USA). GAPDH, β-actin, β-tubulin and PCNA antibodies were employed for the normalization of protein loading.

### 4.4. Cell Culture Treatment

Primary rat renal interstitial fibroblasts (RKFs, purchased from Cellverse Bioscience Technology Co., Ltd., Shanghai, China; Cat#: RAT-iCELL-u014) were cultured in DMEM medium supplemented with 1% fibroblast growth factor (purchased from Cellverse Bioscience Technology Co., Ltd., Shanghai, China), 10% heat-inactivated fetal bovine serum (FBS, Gibco, Waltham, MA, USA) and 1% penicillin-streptomycin, and maintained in an incubator at 37 °C with 5% carbon dioxide (CO_2_). The RKFs were divided into six groups: the CON group, the ALD group (stimulated with 10^−7^ mol/L aldosterone for 24 h), the ALD + ESA group (pretreated with 10^−6^ mol/L esaxerenone 2 h prior to aldosterone treatment), VEGFC group (MCE, Shanghai, China; Cat#: HY-P74474, 100 ng/mL), ALD+ VEGFR-3-IN-1 group (MCE, Shanghai, China; Cat#: HY-P7025, 20 ng/mL; stimulation for 48 h), and VEGFC + VEGFR-3-IN-1 group (MCE, Shanghai, China; Cat#: HY-132305, 20 ng/mL) and stimulated for 24 h.

### 4.5. Flow Cytometry

Following 24 h of drug stimulation, the RKFs were harvested and centrifuged in flow cytometry tubes. The primary anti-VEGFR3 antibody (1:100 dilution; Bioss, Beijing, China, Cat#: bs-2202R) and anti-Prox-1 antibody (1:50 dilution; Proteintech, Chicago, IL, USA, Cat#: 11067-2-AP) were used in combination with either the PE-preabsorbed secondary antibody (1:1000 dilution, Abcam, Shanghai, China, Cat#: ab72465) or CoraLite488-conjugated secondary antibody (1:1000 dilution, Proteintech, Chicago, IL, USA, Cat#: SA00013-2). Subsequently, the cells were incubated with APC-conjugated anti-FSP-1 antibody (2 μL per test, Bioss, Beijing, China, Cat#: bs-3759R) for 1 h. Negative controls were set using unstained cells. Subsequent cell analysis was performed on a BD FACSAria II flow cytometer (BD Biosciences, Franklin Lake, NJ, USA), where live singlet cells were selected through FSC/SSC gating. Data analysis was carried out with FlowJo 10 (USA) software.

### 4.6. Cell Immunofluorescence Staining Assay

Cells were seeded onto dry, sterilized slides placed in 24-well Petri dishes. Following cell adhesion, the cells were fixed with 4% paraformaldehyde at room temperature for 15 min. Subsequently, the cells were treated with 0.25% Triton X-100 for 15 min at room temperature, then blocked with 10% goat serum at 37 °C for 30 min to eliminate non-specific binding. Afterwards, the cells were incubated overnight at 4 °C with primary antibodies against LYVE-1 (1:100, NOVUS, Cat#: 2203R01) and FSP-1 (1:300, ProteinTech, Cat#: 66489-1-Ig). The next day, the cells were incubated with the corresponding secondary antibody for 1 h at 37 °C in the dark, followed by DAPI staining for 10 min to label cell nuclei. Finally, images were captured using an EVOS^®^ FL Auto Cell Imaging System (Thermo Fisher, Waltham, MA, USA).

### 4.7. VEGFR-3 Knockdown and Overexpression in RKFs via Small Interfering RNA (siRNA)

RKFs were seeded into 60-mm culture dishes (DMEM-H + 10%FBS + 1%P/S + 1%FGF), and transfection was initiated when the cell confluence reached 70%. Transfection was performed using Lipofectamine™ 2000 Transfection Reagent (Invitrogen, Carlsbad, CA, USA, Cat#: 11668019) and sample collection and subsequent detection could be conducted after 48 h of transfection. The expression levels of VEGFR-3 and FSP-1 were detected by flow cytometry to evaluate the efficiency of gene knockout and overexpression.

### 4.8. Transcriptome Profiling and Analysis

After 24 h of treatment, the culture medium was aspirated, and the samples were rapidly washed twice with PBS. One milliliter of TRIzol (invitrogen by Thermo Fisher Scientific, Cat No.: 15596018CN, Shanghai, China)reagent was added per 10 cm^2^ of culture area, followed by repeated pipetting of the cells with a 1-mL pipette until no cell clumps were observed and the entire mixture became clear and non-viscous. Total RNA was then extracted from the samples, and the following tests were conducted: determination of RNA purity (OD260/280 and OD260/230 ratios) and precise evaluation of RNA integrity (RIN value). Once the samples passed the quality inspection, a sequencing library was constructed. After the library passed the quality control assessment, Illumina PE150 sequencing was carried out.

### 4.9. RKF Scratch Wound Assay

The bottom of the 6-well plate was marked at 5-mm intervals, and the cells were seeded into the plate. Once the cells were fully adherent, perpendicular scratches were made on the cell monolayer using a sterile 100-µL pipette tip. The plate was gently washed twice with sterile PBS, followed by the addition of stimulation medium. The cells were divided into CON, ALD, and ALD + ESA groups. At 0 h, 6 h, and 12 h after stimulation, RKF migration was photographed under a microscope, and the migration area was analyzed with ImageJ software (Version 2.1.0).

### 4.10. RKF Tube Formation Assay

RKFs were harvested and centrifuged, and the resulting cell pellet was resuspended in DMEM supplemented with 1% penicillin and streptomycin. Suspended cells (2 × 10^6^) were thoroughly mixed with 25 μL of growth factor-reduced Matrigel (BD, USA) and then seeded into a pre-cooled 24-well plate, and incubated in a 37 °C incubator. The cells were observed and photographed using a microscope. The formation of lymphatic vessels was evaluated by measuring the total length of the tubes formed in each image using ImageJ software (version 2.1.0).

### 4.11. Serum Aldosterone Was Quantified by ELISA

Aldosterone levels were detected using an ELISA Kit (Cat No.: E-EL-0070, Elabscience Biotechnology, Wuhan, China). Whole blood samples were kept at room temperature for 1 h or stored overnight at 2–8 °C, followed by centrifugation at 1000× *g* for 20 min at 2–8 °C, and the supernatant was collected for detection. In brief, 50 μL of sample was added to each well, and then 50 μL of biotinylated antibody working solution was immediately added, followed by incubation at 37 °C for 45 min. After discarding the liquid in the wells, the microplate was washed three times. Next, 100 μL of HRP conjugate working solution was added to each well and incubated at 37 °C for 30 min. The plate was washed five times after removing the reaction solution. Subsequently, 90 μL of substrate solution was added and incubated at 37 °C for 15 min. Finally, 50 μL of stop solution was added to terminate the reaction, and the absorbance was measured immediately at 450 nm for subsequent data analysis.

### 4.12. Statistical Analysis

SPSS version 24.0 software (IBM, Armonk, NY, USA) was used for statistical analyses, and GraphPad Prism 8.0 (GraphPad Software, Inc., La Jolla, CA, USA) was used to produce the graphs and figures. Measurement data were presented as mean ± standard deviation (SD). For comparisons among multiple groups of measurement data, normality and homogeneity of variance tests were performed first. If normality and homogeneity of variance were satisfied, one-way ANOVA was used, followed by Tukey’s test for pairwise comparisons. All statistical tests were two-sided, and a *p*-value < 0.05 was considered statistically significant.

## Data Availability

The data supporting the findings of this study are available from the corresponding author upon reasonable request.

## Abbreviations

The following abbreviations are used in this manuscript:

LEC	Lymphatic Endothelial Cell
UUO	Unilateral Ureteral Obstruction
ALD	Aldosterone
RKF	Rat Kidney Interstitial Fibroblast
CKD	Chronic Kidney Disease
FSP-1	Fibroblast Specific Protein-1
MR	Mineralocorticoid Receptor
ESA	Esaxerenone
Ccr	Creatinine Clearance Rate
MCP-1	Monocyte Chemotactic Protein-1
TNF-α	Tumor Necrosis Factor-Alpha
IL-1β	Interleukin-1β
VEGFC	Vascular Endothelial Growth Factor C
VEGFR-3	Vascular Endothelial Growth Factor Receptor 3
LYVE-1	Lymphatic Vessel Endothelial Hyaluronan Receptor 1
